# Phytodepuration of Nitrate Contaminated Water Using Four Different Tree Species

**DOI:** 10.3390/plants10030515

**Published:** 2021-03-10

**Authors:** Luca Regni, Maria Luce Bartucca, Euro Pannacci, Francesco Tei, Daniele Del Buono, Primo Proietti

**Affiliations:** Department of Agricultural, Food and Environmental Sciences, University of Perugia, Via Borgo XX Giugno 74, 06121 Perugia, Italy; luca.regni@unipg.it (L.R.); marialucebartucca@gmail.com (M.L.B.); euro.pannacci@unipg.it (E.P.); francesco.tei@unipg.it (F.T.); primo.proietti@unipg.it (P.P.)

**Keywords:** phytoremediation, tree species, nitrate pollution, buffer strips, *Salix alba* L., *Populus alba* L., *Corylus avellana* L., *Sambucus nigra* L.

## Abstract

Water pollution by excessive amounts of nitrate (NO_3_^−^) has become a global issue. Technologies to clean up nitrate-contaminated water bodies include phytoremediation. In this context, this research aimed to evaluate four tree species (*Salix alba* L., *Populus alba* L., *Corylus avellana* L. and *Sambucus nigra* L.) to remediate nitrate-contaminated waters (100 and 300 mg L^−1^). Some physiological parameters showed that *S. alba* L. and *P. alba* L. increased particularly photosynthetic activity, chlorophyll content, dry weight, and transpired water, following the treatments with the above NO_3_^−^ concentrations. Furthermore, these species were more efficient than the others studied in the phytodepuration of water contaminated by the two NO_3_^−^ levels. In particular, within 15 days of treatment, *S. alba* L. and *P. alba* L. removed nitrate quantities ranging from 39 to 78%. Differently, *C. avellana* L. and *S. nigra* L. did not show particular responses regarding the physiological traits studied. Nonetheless, these species removed up to 30% of nitrate from water. In conclusion, these data provide exciting indications on the chance of using *S. alba* L. and *P. alba* L. to populate buffer strips to avoid NO_3_^−^ environmental dispersion in agricultural areas.

## 1. Introduction

Nowadays, many anthropogenic activities release large quantities of organic and inorganic substances into the environment, posing severe dangers to aquatic habitats, animal and human health [1,2]. In particular, industries introduce vast quantities of compounds into the environment [2]; additionally, wastewater disposal has a considerable negative influence on the environment [2]. Concerning agriculture, this activity substantially impacts the environment [3]. Indeed, intensive agriculture, aimed at achieving high yields and crop productivity, can create several environmental dangers and, in particular, negatively alter the status of water resources [4]. Finally, the world growing population will require ever-increasing quantities of food in the coming years, thus raising the pressure on cropping systems and, in turn, on the environment [5].

Nitrogen is among the substances routinely employed in agriculture, causing more than one concerns. In particular, it is applied in massive amounts as fertilizers in farming systems [6,7], and for its chemical and physical properties can, through run-off, reach freshwaters [8]. High concentrations of nitrogen dispersed into the environment can lead to freshwaters’ eutrophication, with very adverse effects on aquatic ecosystems [9].

The most common form of nitrogen of anthropogenic provenience in water is nitrate (NO_3_^−^). NO_3_^−^ can be found in the natural background at levels ranging from 0 to 2.0 mg L^−1^ [4]. However, in areas with intensive agriculture or densely populated, especially in developing countries, its concentration can be in the water more than 100 times higher [10]. Particularly worrying are the data recorded in some regions of the planet where nitrate concentrations can easily exceed 100 mg L^−1^ or even reach up to 500 mg L^−1^ [11,12,13]. Contamination of freshwater by nitrate represents an immediate risk to human health, especially when it reaches drinking water. For this purpose, the World Health Organisation (WHO) stated that the nitrate concentration admitted to drinking water should not exceed 50 mg L^−1^ [11]. Indeed, human consumption of drinking water with higher concentrations of NO_3_^−^ can cause illness, such as blue disease in babies (methemoglobinemia) [14].

Some different technologies allow us to remediate polluted environments. Among these, phytodepuration has become particularly popular in recent years [15]. This technology uses plant species showing appropriate physiological and biochemical characteristics to remove toxic substances or excessive amounts of nutrients from contaminated water and soil [2]. The plant’s ability to remediate contaminated water is due to its capacity to cope with the potential toxicity that the substance to be removed from polluted sites could exert and on its potential to accumulate and metabolize it [15].

As for the phytodepuration of water from nitrate, several species have been considered in recent studies for their ability to act as filters, thus absorbing and trapping, among other things, nitrogen compounds, which are mainly present in the form of nitrate. In these studies, crops, grasses, ornamental and aquatic species have been investigated. In particular, *Chrysopogon zizanioides*, *Utricularia aurea*, *Vetiveria zizanioides*, *Oryza sativa*, *Epiprennum aureum*, *Syngonium podophyllum*, *Eichhornia crassipes*, *Panicum hemitomon*, *Echinodorus cordifolius*, *Myriophyllum aquaticum*, *Typha latifolia*, *Saururus cernuus*, *Lolium multiflorum*, *Ranunculus nipponicus*, and *Pistia stratioes* showed great results when applied to remediate contaminated aquatic samples [9,14,16,17,18,19,20,21,22].

In this context, tree species in phytoremediation programs are attracting increasing attention during the last years. These plants can show some characteristics which make them suitable and intriguing for this technology, especially in synergy with herbaceous plants due to different root system depths. The fast growth of some woody species, the particular and deep root system, and the dense branches have been considered good traits to remediate polluted environments [23]. Despite this, trees have been mainly studied for the decontamination of environments polluted by heavy metals. They have been found to efficiently remove harmful species such as ionic Cd, Cu, Hg, Pb, Cr, Fe, and Zn [23,24,25,26].

As for the nitrate, transgenic poplar trees have been employed to clean water polluted from amounts of this substance up to twenty times higher than that admitted by the European Union [27]. Additionally, Warsaw et al. [28] investigated *Salix alba* and *Sambucus nigra* to remove two herbicides (metalaxyl and trifluralin) and nitrate in aquatic zones. All these authors found satisfying remediating capacities for the trees studied. Despite this, it is evident that little research has been carried out to assess whether tree species can remedy water contaminated by excessive amounts of nitrate. For this reason, this topic remains an open and interesting field of study, considering its possible applications and implications.

For the reasons mentioned above, the present work aims to evaluate the phytodepuration potential of four tree species common along rivers in Italy *Salix alba* L., *Populus alba* L., *Corylus avellana* L. and *Sambucus nigra* L. and detect any differences between these species to decontaminate the water from nitrate. To this end, the experiments were carried out in hydroponic, as this system is useful for this type of study, and, even though it is different from the open field, it allows comparing different species in uniform conditions, avoiding the interferences due to the soil types and microbiota. Finally, the data acquired helps understand the potential of the tree species to accumulate toxic compound. Therefore, the results obtained can, in turn, provide useful information on the choice of the species most suitable for vegetating buffer strips.

## 2. Results

### 2.1. Leaf Net Photosynthesis (Pn) and Chlorophyll Content

During the experimental period, significant increases in photosynthetic rates were found for *Populus* and *Salix* in nitrate-treated plants at 15 days after treatment (DAT), while in *Corylus* and *Sambucus* increases in photosynthetic rate were found only at the highest dosage of nitrate (Table 1). SPAD (Soil Plant Analysis Development) values showed in both *Populus* and *Salix* an increase in chlorophyll content in plants grown in nitrate contaminated water, starting at 7 DAT, compared with control plants, with a greater effect of treatment at the 300 mg L^−1^ of NO_3_^−^ compared with 100 mg L^−1^ of NO_3_^−^ (Table 2). In *Corylus* and *Salix* increases in SPAD values were observed only at 15 DAT and at 300 mg L^−1^ dosage.

### 2.2. Plant Growth and Evapotranspirated Water

Table 3 shows the dry weight recorded for the four plants investigated and subjected to the treatment with 100 and 300 mg L^−1^ compared to controls samples. *Salix* and *Populus* showed that the nitrate treatments generally increased plant dry weight (DW), and this effect was more pronounced at 300 mg L^−1^. The increase in plant DW was mainly due to an increase in stem+shoots and leaves DW. On the contrary, the amount of nitrate did not affect dry weight in *Corylus* and *Sambucus*. Generally, the aboveground/belowground DW ratio increased in all species at 300 mg L^−1^.

The amount of evapotranspirated water (Table 4) cumulated after 21 DAT generally increased at 100 and 300 mg L^−1^ of NO_3_^−^, although with varying intensities depending on the species. In particular, a clear increase in transpired water in the plants grown in nitrate contaminated water was observed in *Salix* and Populus. On the contrary, in *Sambucus* and *Corylus* no differences were observed for the transpired water between control plants, and samples treated with 100 and 300 mg L^−1^ of NO_3_^−^.

### 2.3. Nitrate Absorption

**Figure 1:**
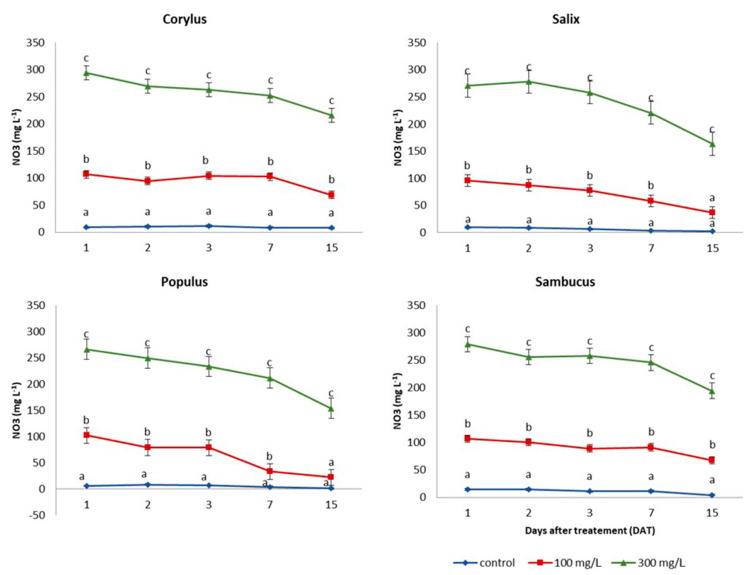
Nitrate concentration of nutrient solutions at 1, 2, 3, 7, and 15 days after treatment (DAT) with NO_3_^−^ in *Corylus*, *Salix*, *Sambucus* and *Populus*. For each time point and each species, values followed by the different letter are significantly different at *p* < 0.05.

*Populus* and *Salix* plants were found very efficient to reduce nitrate in water over the experimental period (Figure 1). In fact, after 21 DAT, the *Populus* removed about 78% and 42% for the dosage 100 mg L^−1^ at the 300 mg L^−1^, respectively. In *Salix*, the higher absorption (61%) was observed at 100 mg L^−1^, while, at 300 mg L^−1^, the nitrate reduction was equal to 39%. *Corylus* and *Sambucus* plants at 15 DAT were less effective in reducing the nitrate concentration, which was found around 30% at the doses of 100 and 300 mg L^−1^ of NO_3_^−^. However, at 100 mg L^−1,^ all the species used, except *Sambucus*, restored a good quality of the water with a nitrate concentration below 50 mg L^−1^. In contrast, nitrogen consumption in control plants was relatively modest (around 15%) during the period considered.

### 2.4. Bioconcentration Factor (BCF)

Table 5 shows the bioconcentration factor (BCF) exhibited by the four species investigated in this study concerning the amount of NO_3_^−^ subministrated to the growth media. BCF is indicative of the plant ability to accumulate nitrate from the surrounding environment. The table also shows the BCF values exhibited by the species grown in solutions containing the proper amount of nitrate (control samples). In this specific case, *Populus* and *Salix* showed the highest BCF values. However, when examining the BCF results, the concentrations 100 and 300 mg L^−1^ should be considered since they are representative of cases of nitrate contamination. *Populus* showed the highest BCF values, reaching values of 0.72 and 2.43 for concentrations of NO_3_^−^ of 100 and 300 mg L^−1^, respectively. The other species investigated in this research showed lower BCF values. In particular, *Salix* had a BCF higher than the unity when treated with 100 mg L^−1^ nitrate. In contrast, *Corylus* and *Sambucus* exhibited similar BCF values at the two dosages investigated (100 and 300 mg L^−1^), which were 0.43.

## 3. Discussion

The problem of environmental pollution due to the dispersion of nitrate in surface water or groundwater is very topical. In this context, the study of plant species capable of tolerating even high nitrate quantities for environmental remediation is essential. To date, lesser attention has been paid to tree plants than to herbaceous species for phytoremediation studies. Furthermore, although many studies have been done on plant species to understand NO_3_^−^ removal [29,30,31], the mechanisms involved in this process remain little known [32]. For the reasons mentioned above, the present study was planned to obtain data and understand if *Salix*, *Populus*, *Corylus*, and *Sambucus* could be considered suitable to remediate water contaminated by nitrate levels well above the admitted limits [15]. Furthermore, this study aimed to understand the plant responses during phytodepuration by investigating different physiological and biometric parameters.

*Salix* and *Populus’* photosynthesis and chlorophyll content (expressed as SPAD index) increased at both the dosages of nitrate used (Table 1 and Table 2). The increase in chlorophyll content showed mainly by *Populus* and *Salix* treated with nitrate at 100 and 300 mg L^−1^ can be reasonably explained based on the fact that the N is essential for chlorophyll biosynthesis [9]. Thus, the increase for the two species in chlorophyll content indicates that nitrogen at these concentrations does not lead to toxic effects and, on the contrary, represents a plant-usable nitrate intake from the growth medium [33]. Moreover, chlorophyll content, photosynthesis and respiration rates are closely linked to the N concentration, and, in turn, increases in N content would up-regulate carbon metabolism [34]. *Salix* and *Populus* showed significant increases in photosynthetic activity, particularly at 15 days after the treatments, while *Corylus* and *Sambucus* showed an increase in photosynthetic activity only at the highest dosage of nitrate applied to the growth media.

The study of plant dry matter and evapotranspirated water, which are related to the photosynthetic activity, which in turn depends on the leaf chlorophyll content, provided essential indications of a good response exhibited by *Populus* and *Salix* following the nitrate treatments (Table 3 and Table 4). In fact, it is well-known that nitrogen is an essential nutrient for plants growth and development, being indispensable for protein synthesis and plant metabolism [35]. Furthermore, nitrate is the nitrogen-preferred form by plants grown without soil [34]. It has been shown that increases in dry weight can be observed, increasing the nitrogen load in the growth medium [34]. However, this response is depending on the species considered [9,36]. Despite this, our results agree with this species-dependent behavior, showing that *Populus* and *Salix* exhibited significant dry weight increases. These results align with the increases in photosynthesis and chlorophyll observed for *Salix* and *Populus* (Table 3) since the biomass production is strictly related to the photosynthetic activity. Nonetheless, the biomass increases resulted from a higher dry weight of stem and shoots for *Salix* and stem, shoots and leaves for *Populus*. In contrast, the other two species studied were unaffected by the dosages used in this experimentation. Our findings can seem apparently in contrast with other results. For instance, Bravo and Hill [37] found that high nitrate concentration in the groundwater did not influence white cedar (*Thuja occidentalis*) tree growth. However, as evidenced by the four species studied, the plant capacity to uptake nitrate is strictly species-depending. Finally, it should be emphasized that one of the preferred requirements of plants for phytoremediation is fast growth and rapid accumulation of biomass [2]. Therefore, *Salix* and *Populus* provided positive indications in this respect.

Transpiration is a physiological trait very sensitive to stresses of various kinds and correlates to proper stomatal activity [38,39]. If transpiration is combined with plant biomass development, it provides useful information on its health status [40]. Furthermore, in some cases, the transpiration increases have been positively correlated with crop production following high nitrate levels in the growth medium [41]. In particular, nitrate increases can improve transpiration without adverse effects on the species in question, leading, on the contrary, to increases in biomass [41]. Our experiments are in line with these sherds of evidence for *Populus* and *Salix*. In fact, these species showed increases in evapotranspiration and biomass in response to both the nitrate dosages studied.

The primary mechanisms for nitrate removals in buffer strips are vegetation uptake and microbial denitrification [42]. The role of the vegetation is still not fully understood, and often the results are in conflict. Considerable errors in estimating the relative importance of plant uptake and denitrification can occur [43]. In some researches, the vegetation has no significant effect on nitrate uptake [44,45,46,47,48]. On the contrary, Clement et al. [49] and Donth et al. [50] found that plant’s nitrate removal occurred mainly in high water table periods. In contrast, plant nitrate uptake was not significant in the summer when the water table declines, and denitrification plays the leading role. Borin and Bigon [51] showed significant nitrate removal rates (around 90%) with a 5 m buffer strip composed of herbaceous species and a row of deciduous tree species. The uptake data of about 70-80% observed in the present study for *Populus* agree with Donth et al. [50] that found a nitrate depletion equal to 75%, but, in this last case, the removal was attributed to the denitrification process.

In our study, *Populus* and *Salix* exhibited the highest remediating capacity for the nitrate concentrations investigated (Figure 1). However, all the tested species showed attractive abatement rates of the nitrate that, at the beginning of the experiment, was below the quality standard of groundwater of 50 mg L^−1^, which is the value specified in the European Union Groundwater Directive (2006/118/EC). The initial nitrate uptake exhibited by the species investigated is worth mentioning. In particular, as shown in Figure 1, plants absorbed nitrate very slowly in the first days after the treatments. On the last days of the trial, a more rapid uptake was observed. This dynamic is well documented in the literature. It depends on the fact that nitrate influx in plants, as its concentration increases, is progressively regulated by inducible transport systems. Glass et al. [52] reported in their studies that this activation time could take around three days in certain species.

The bioconcentration factor (BCF) indicates the plant’s effectiveness in removing a pollutant from a contaminated environment [53]. BCF is the ratio of the pollutant picked up by the plant and that remained in the substrate. BCF is used to screen plants for their potential use in phytoremediation. Plants showing a BCF close to the unity or higher may have a significant potential for the phytoremediation of a given contaminant [9]. BCF decreases with increasing contaminant concentrations can be due to toxicity effects or regulatory mechanisms that control and decrease nitrate influx to protect the species [9]. Values closed to the unity were found in *Populus* at the highest nitrate dose of 300 mg L^−1^ and much higher than unity at 100 mg L^−1^ (Table 5). *Salix* showed a value well above unity at this last dose of nitrate. The other plants studied did not show significant phytoremediating potential based on the BCF values.

These results of nitrate uptake from water and BCF indicate that *Salix* and *Populus*, concerning the concentration of NO_3_^−^ in the growth medium, were the most suitable tree species, among those investigated, to remediate contaminated waters. From an application standpoint, this study suggests *Salix* and *Populus* suitable for vegetating buffer strips, which are uncultivated zones left in agricultural areas’ boundaries to avoid contaminant dispersion into the environment [15]. *Populus* has been already indicated suitable for such an application since this species has a high transpiration rate and suitable capacity to absorb, degrade and/or detoxify contaminants, showing a great affinity for nitrate [54].

## 4. Materials and Methods

### 4.1. Plant Material, Hydroponic System, and Nitrate Treatment

Rooted cuttings with 15 cm average height of *Salix alba* L. (#Salix), *Populus alba* L. (#Populus), *Corylus avellana* L. (#Corylus) and *Sambucus nigra* L. (#Sambucus) were used. They were removed from the perlite of the mist propagation system and, after root washing with distilled water, placed in 800 mL pots containing expanded clay and put under hydroponic conditions for an adaptation period of 30 days. The recirculating hydroponic solution was composed of a half-strength Hoagland solution (pH 7.5). The hydroponic system is composed of PVC containers. Each container includes five plastic hydroponic pots, and it is connected to a tank (volume 3.5 l each) containing the nutrient solution. An automated system ensured the nutrient solution’s flux from the tank to the PVC containers three times per day. The hydroponic system was maintained in a growing chamber, and plants were exposed to daylight with an active photosynthetic radiance by a system equipped with lamps (PHILIPS SON-T AGRO 400 W, Amsterdam, Netherlands) producing 200 μmol m^−2^s^−1^ photon flux density, under a photoperiod of 16 h d^−1^. The temperature was set at 23 °C (+/−1 °C) and relative humidity at about 60%. The nutrient solution was replaced every 30 days. At the end of the adaptation period, three tanks for each species (15 plants for each species) were left as controls (NO_3_^−^ concentration 7 ± 3.4 mg L^−1^ and referred as “0”). In three tanks for each species, KNO_3_ was added to a NO_3_^−^ concentration of 100 mg L^−1^. Additionally, in three tanks for each species, KNO_3_ was added to a concentration of 300 mg L^−1^. The nitrate concentrations used were double and six-fold higher than the limit (50 mg L^−1^) established in the European Union Groundwater Directive (2006/118/EC). Furthermore, these NO_3_^−^ concentrations selected in this study cover the amount of nitrate found in some rivers of the planet [11,12,13,55].

### 4.2. Leaf Net Photosynthesis (Pn) and Chlorophyll Content

Leaf net photosynthesis (Pn) was determined on six leaves for each treatment at 1, 7, and 15 DAT using a portable IRGA (ADC-LCA-3, Analytical Development, Hoddesdon, UK) and a Parkinson-type assimilation chamber. Leaves were enclosed in the chamber and exposed to the same light as in the hydroponic system. The flow rate of air passing through the chamber was kept at 5 cm^3^ s^−1^. During gas-exchange measurements, the external CO_2_ concentration was about 375 cm^3^ m^−3^, and the air temperature inside the leaf chamber was 1 °C higher than the hydroponic room temperature. Measurements were taken under steady-state conditions (after about 30 s). Pn was expressed on a leaf area basis. The chlorophyll content was measured on twelve leaves for each treatment at 1, 7 and 15 DAT by a SPAD-502 chlorophyll meter (Minolta Camera Co. Ltd., Sakai, Osaka Japan,).

### 4.3. Evapotranspiration and Plant Growth

The water lost through evapotranspiration was recorded daily, measuring the amount of water required to bring the nutrient solution volume to 3.5 L. At the end of the experiment, 21 days after the NO_3_^−^ addition (DAT), six plants for each treatment were selected, and roots, shoots, stems and leaves of each plant were weighed fresh (FW) and then oven-dried at 95 °C until a constant weight was reached, to determine the dry weight (DW).

### 4.4. Nitrate Absorption

At 1, 2, 3, 7, and 15 days after treatment, a proper water volume (30 mL) was taken from each tank and analyzed for nitrate concentration according to the methods described in [9]. In particular, 1 mL of sodium salicylate was added to a sample of 10 mL of water and evaporated in a glass capsule. Then, 1 mL of concentrated sulphuric acid was added to the residue. 10 mL of Seignette salt and 10 mL of sodium hydroxide were added and mixed. The absorbance was immediately recorded with a spectrophotometer at the wavelength of 450 nm. A calibration curve was constructed by preparing a series of standards from the potassium nitrate solution. The result is expressed in mg L^−1^ of N (1 mg L^−1^ of N = 4.4 mg NO_3_^−^).

### 4.5. Bioconcentration Factor (BCF)

Bioconcentration factor at 15 DAT was calculated according to Zhuang et al. [28] as follows:

BCF = C_harvested tissue_/C_substrate_

where C_harvested_ tissue is the concentration of the target pollutant in the plant harvested tissue. C_subsrate_ is the concentration of the same compound in the hydroponic nutrient solution. The difference between the initial amount of nitrate added to the water and the residue found at the end of the experiments allowed us to estimate the NO_3_^−^ absorbed by plants [9]. This assumption was validated by monitoring NO_3_^−^ concentrations over time in some containers without the plant species. The amount of nitrate did not change over time in the absence of plants.

### 4.6. Statistical Analysis

The trials were organized according to a randomised block design, with three treatments and three replications per treatment (15 plants for each treatment). Statistical analysis was performed by analysis of variance (ANOVA). Significant differences between the values were determined at *p* ≤ 0.05, according to the Tukey HSD method.

## 5. Conclusions

All the species used exhibited an interesting nitrate absorption, but, among them, the *S. alba* L. and *P. alba* L. showed the highest capacities to remove nitrate from water, reducing its concentration of values in the range 39–78%. More precisely, *P. alba* L. seems to be the one with the best capacity to absorb nitrate and exhibits the highest BCF. Finally, nitrate determined in *S. alba* L. and *P. alba* L. higher leaves chlorophyll contents and photosynthetic rates. Consequently, these effects increased plant dry weight and transpiration rate. Therefore, this research indicated these two species as suitable plants for creating buffer strips, zones around cultivated areas, which prevent, among other things, nitrate’s environmental dispersion.

Additionally, this study showed no toxic effects of nitrate on plants. This may result from defensive mechanisms activated by the plants or, as generally considered in the case of nitrate, from the species’ ability to regulate and control its acquisition, thus avoiding excessive loads. However, another factor to be considered is the exposure time of these experiments, which may have had a decisive effect in avoiding the onset of toxicity effects.

However, to better delineate the phytodepurative capacity of tree species, further studies on the mechanisms of nitrogen transformation in plants are needed. The knowledge of biochemical mechanisms and response to high amounts of nitrate will fully exploit plants’ potential to remediate nitrate.

## Figures and Tables

**Table 1:** Leaf net photosynthesis (Pn) of the leaves of *Corylus*, *Salix*, *Sambucus*, and *Populus* treated with the three NO_3_^−^ concentrations in hydroponic nutrient solution at 1, 7, and 15 days after the treatment (DAT).

	1 DAT µmol CO_2_ m^−2^ s^−1^	7 DAT µmol CO_2_ m^−2^ s^−1^	15 DAT µmol CO_2_ m^−2^ s^−1^
Corylus-0	1.84 ± 0.23 a	3.12 ± 0.41 a	2.64 ± 0.34 a
Corylus-100	2.12 ± 0.19 a	2.32 ± 0.27 a	2.23 ± 0.45 a
Corylus-300	2.05 ± 0.25 a	2.95 ± 0.34 a	3.92 ± 0.56 b
Salix-0	2.61 ± 0.37 a	2.12 ± 0.38 a	2.63 ± 0.37 a
Salix-100	2.72 ± 0.29 a	3.36 ± 0.54 a	4.60 ± 0.43 b
Salix-300	2.36 ± 0.42 a	2.34 ± 0.59 a	4.67 ± 0.39 b
Sambucus-0	2.43 ± 0.41 a	2.09 ± 0.37 a	1.90 ± 0.42 a
Sambucus-100	1.95 ± 0.34 a	2.48 ± 0.24 a	2.25 ± 0.51 a
Sambucus-300	2.05 ± 0.45 a	1.82 ± 0.21 a	4.10 ± 0.63 b
Populus-0	2.45 ± 0.23 a	2.79 ± 0.38 a	2.63 ± 0.31 a
Populus-100	2.39 ± 0.31 a	4.28 ± 0.57 a	5.18 ± 0.89 b
Populus-300	3.30 ± 0.29 a	3.50 ± 0.34 a	5.33 ± 0.92 b

Means in each column ± SE for each species followed by the different letter are significantly different at *p* < 0.05.

**Table 2:** Chlorophyll content (expressed as SPAD measurements) of the leaves of *Corylus*, *Salix*, *Sambucus*, and *Populus* grown treated with the three NO_3_^−^ concentrations in hydroponic nutrient solution at 1, 7, and 15 days after the treatment (DAT).

	1 DAT	7 DAT	15 DAT
Corylus-0	25.63 ± 3.74 a	27.26 ± 3.24 a	25.78 ± 2.01 a
Corylus-100	27.14 ± 4.15 a	26.54 ± 2.21 a	24.13 ± 1.87 a
Corylus-300	25.14 ± 3.95 a	25.64 ± 3.16 a	29.36 ± 1.41 b
Salix-0	28.84 ± 2.75a	31.12 ± 2.77 a	30.72 ± 1.47 a
Salix-100	27.56 ± 4.32 a	38.54 ± 3.24 b	34.47 ± 2.13 b
Salix-300	29.25 ± 3.12 a	37.47 ± 3.12 b	36.07 ± 3.42 b
Sambucus-0	26.32 ± 3.13 a	24.78 ± 4.13 a	25.47 ± 4.32 a
Sambucus-100	27.12 ± 4.84 a	25.63 ± 3.45 a	26.34 ± 3.15 a
Sambucus-300	26.83 ± 2.98 a	27.54 ± 2.23 a	30.27 ± 2.76 b
Populus-0	27.74 ± 5.23 a	26.15 ± 2.13 a	28.86 ± 2.57 a
Populus-100	28.10 ± 3.21 a	31.23 ± 2.21 b	34.00 ± 1.94 b
Populus-300	26.65 ± 2.72 a	32.11 ± 2.05 b	36.07 ± 2.34 b

Means in each column ± SE for each species followed by the different letter are significantly different at *p* < 0.05.

**Table 3:** Dry weight (DW) of different parts and total DW of plants of Corylus, Salix, *Sambucus* and *Populus* treated with the three NO_3_^−^ concentrations in hydroponic nutrient solution at 21 days after the treatment (DAT).

	DW Roots	DW Stem + Lateral Shoots	DW Leaves	DW Total
	(g)	(g)	(g)	(g)
Corylus-0	1.97 ± 0.43 a	1.99 ± 0.43 a	1.22 ± 0.17 a	5.18 ± 0.54 a
Corylus-100	1.84 ± 0.59 a	2.00 ± 0.51 a	1.31 ± 0.18 a	5.15 ± 0.61 a
Corylus-300	1.09 ± 0.31 a	2.56 ± 0.32 a	0.86 ± 0.12 a	4.51 ± 0.53 a
Salix-0	0.37 ± 0.15 a	2.60 ± 0.32 a	0.57 ± 0.11 a	3.54 ± 0.38 a
Salix-100	0.41 ± 0.18 a	2.80 ± 0.27 a	0.79 ± 0.13 a	4.00 ± 0.55 a
Salix-300	0.37 ± 0.13 a	4.46 ± 0.72 b	1.05 ± 0.24 a	5.88 ± 0.43 b
Sambucus-0	0.43 ± 0.17 a	2.12 ± 0.43 a	0.53 ± 0.14 a	3.08 ± 0.32 a
Sambucus-100	0.31 ± 0.12 a	2.16 ± 0.37 a	0.53 ± 0.17 a	3.00 ± 0.27 a
Sambucus-300	0.42 ± 0.17 a	1.43 ± 0.22 a	0.44 ± 0.12 a	2.29 ± 0.32 a
Populus-0	0.28 ± 0.14 a	3.42 ± 0.31 a	0.54 ± 0.10 a	4.24 ± 0.45 a
Populus-100	0.47 ± 0.18 a	3.23 ± 0.37 a	1.72 ± 0.23 b	5.42 ± 0.33 b
Populus-300	0.41 ± 0.15 a	4.68 ± 0.43 b	1.67 ± 0.31 b	6.76 ± 0.41 b

Means in each column ± SE for each species followed by the different letter are significantly different at *p* < 0.05.

**Table 4:** Cumulated evapotranspirated water by Corylus, Salix, *Sambucus* and *Populus* treated with the tree NO_3_^−^ concentrations in hydroponic nutrient solution at 21 days after the treatment (DAT).

	Evapotranspirated H_2_O
	(mL)
Corylus-0	2.550 ±545 a
Corylus-100	2.492 ± 314 a
Corylus -300	2.104 ± 274 a
Salix-0	3.296 ± 412 a
Salix-100	4.494 ± 719 b
Salix-300	4.730 ± 516 b
Sambucus-0	1.544 ± 187 a
Sambucus-100	1.930 ± 210 a
Sambucus-300	1.735 ± 197 a
Populus-0	2.471 ± 214 a
Populus-100	3.758 ± 423 b
Populus-300	3.984 ± 456 b

Means in each column ± SE for each species followed by the different letter are significantly different at *p* < 0.05.

**Table 5:** Bioconcentration factor (BCF) in *Corylus*, *Salix*, *Sambucus*, and *Populus* calculated for the four tree species based on the amount of nitrate absorbed from the solutions at 15 DAT.

	BCF
Corylus-0	0.30 ± 0.13
Corylus-100	0.43 ± 0.11
Corylus-300	0.43 ± 0.12
Salix-0	2.61 ± 1.31
Salix-100	1.56 ± 0.18
Salix-300	0.64 ± 0.14
Sambucus-0	1.52 ± 0.57
Sambucus-100	0.43 ± 0.15
Sambucus-300	0.43 ± 0.16
Populus-0	6.30 ± 2.45
Populus-100	2.43 ± 0.31
Populus-300	0.72 ± 0.17

## Data Availability

Data sharing is not applicable to this article.
